# Reconstructing Real-World Vehicle Side-Impact Accidents to Computationally Investigate Far-Side Occupant Injury Risk

**DOI:** 10.3390/biomimetics11020126

**Published:** 2026-02-09

**Authors:** Sha Deng, Ke Peng, Jing Zhang, Danqi Wang, Fang Wang

**Affiliations:** 1 School of Economics and Management, Hunan Open University, Changsha 410004, China; dengsha@hnou.edu.cn; 2 College of Mechanical and Vehicle Engineering, Changsha University of Science and Technology, Changsha 410114, China; 22103020654@stu.csust.edu.cn (K.P.); danqi_wang@csust.edu.cn (D.W.); 3 Hunan Province Key Laboratory of Safety Design and Reliability Technology for Engineering Vehicle, Changsha University of Science and Technology, Changsha 410114, China; 4 Sany Automobile Manufacturing Co., Ltd., Changsha 410100, China; zhangjing4@sany.com.cn

**Keywords:** side-impact collision accident, far-side occupant, finite element model, head injury, chest injury, biomimetics, biofidelic modeling

## Abstract

In side-impact collisions, the occupant in the non-impacted far-side position faces a high risk of death and serious injury. However, current research on injury to far-side occupants remains limited. This study utilized 40 real-world side collision cases to extract dynamic boundary condition parameters of the impacted vehicle through kinematic reconstruction. These parameters were input into a simplified finite element (FE) vehicle model equipped with a human body FE model in the far-side position. Simulation calculations were performed to obtain head and chest injury parameters for the far-side occupant and assess their injury risk. Finally, the study focused on analyzing the effect of vehicle motion boundary conditions on far-side occupant’s injury risk. The assessment based on the head injury criterion HIC_15_ shows a low head injury risk for the far-side occupant. However, using the BrIC metric, which accounts for head rotational motion, reveals a significant risk of severe traumatic brain injury in some cases. Regarding chest injury, analysis based on the effective plastic strain of ribs indicated a low risk of rib fractures. However, results from the chest viscosity criterion (VC) and internal organ strain analysis suggested a high risk of soft tissue injury in the chest. This computational investigation, leveraging biofidelic human models, underscores that the human body’s response to complex, multi-directional impacts is not fully captured by traditional metrics. This study concludes that addressing the protection of the far-side occupant is essential in side-impact safety design, with particular emphasis on the unique injury risks posed by vehicle rotational motion, potentially inspiring biomimetic safety systems that better adapt to these complex loading conditions.

## 1. Introduction

Side collisions are a common type of road traffic accident, accounting for as much as 41% of all crash types and ranking among the most frequent accident categories [1]. Furthermore, side collisions contribute to 25% to 35% of all severe injuries resulting from vehicle collisions [2]. Side collisions can be classified as near-side or far-side based on their impact location. Statistical analysis of side-impact accidents in the U.S. NASS-CDS database indicates that far-side collisions constitute 9.5% of all side-impact crashes [3,4]. Ryb et al. conducted a comparative analysis of occupant fatality and serious injury rates in near-side versus far-side positions across 380 collision cases from the Collision Injury Research and Engineering Network (CIREN) database. They found that 72% of fatalities and severe injuries occurred in the near-side position, while 28% occurred in the far-side position [5]. This indicates that occupant injuries in the far-side position are a significant concern.

The injury patterns and risks for far-side occupants differ from those of near-side occupants [6,7]. Far-side occupants share similarities with near-side occupants, and head and chest injuries remain the primary causes of injury or death in far-side crashes [3,8,9,10]. Moreover, there is no significant difference between near-side and far-side occupants in the risk of sustaining MAIS 3+ severe head injuries (MAIS stands for Maximum Abbreviated Injury Scale. The AIS is currently the most widely used indicator for quantifying the severity of human injury, with AIS 0 and AIS 6 corresponding to no injury and death, respectively [11]). However, far-side occupants face a greater risk of head injury when not restrained by seat belts [5]. Forman et al. conducted side and oblique side (90° and 60° test angles) sled crash tests using postmortem human subjects (PMHS), finding that the 90° angle collision resulted in greater lateral head displacement for far-side occupants [4,12]. Additionally, far-side occupants experienced higher rates of secondary collisions with contralateral structures and near-side occupants, leading to increased head injury. This injury mechanism differs entirely from that of the near-side occupants, for which the intrusion of the struck side structure is the main cause of injury. Fildes et al. [13] analyzed the probability of AIS 3 injury across all body regions for the far-side occupant, finding that chest injuries were most frequent. Rib fractures and pulmonary injuries were the most common forms, accounting for over 80% of AIS 3 chest injuries. Over two-thirds of far-side occupant injuries stem from collisions with the struck-side interior trim, seat belts or buckles, adjacent seats, or the center console [3].

Early research on far-side occupants primarily relied on PMHS to obtain kinematic and injury responses of various far-side body regions during side collisions [4,12,14,15]. However, due to ethical constraints and other factors, conducting cadaver studies presents significant challenges. Researchers have attempted to use side-impact dummies (such as WorldSID, ES-2, and SID-IIs) to investigate injuries sustained by far-side occupants in vehicle side collisions [16,17,18]. Yet existing dummy technologies were developed for protecting near-side occupants and cannot accurately simulate the biofidelic response of far-side occupants [4,19,20]. Meanwhile, mainstream side-impact occupant protection test protocols uniformly employ a moving deformable barrier (MDB) to strike specific locations on the vehicle’s side at speeds between 50 km/h and 60 km/h, striking at a 90° angle to the vehicle’s center plane [21]. The vehicle side-impact safety performance is then evaluated using injury metrics from the side-impact dummy. However, collision speed, angle, and impact location are highly variable in real-world vehicle side-impacts. As demonstrated by Bahouth et al. [22], the most frequent collision angle resulting in AIS 3+ far-side occupant injuries in side impact accidents is 60°. At the same time, these regulations and testing protocols focus solely on injury to near-side occupants in the collision, without evaluating the injury protection performance for far-side occupants. Although NCAP programs (such as Euro-NCAP and C-NCAP) have begun addressing this issue in recent years, their testing scenarios remain highly limited and restricted [23].

In summary, current research on far-side occupant injury in side collisions remains limited to statistical analysis of traffic accident data, cadaver studies under typical conditions, and numerical simulations. There has been insufficient in-depth investigation into the injury mechanisms of far-side occupants in real-world side collision scenarios, which has hindered the advancement of vehicle safety assessment protocols targeting protection for far-side occupants. Meanwhile, numerical studies on far-side occupants have neither performed high-fidelity reconstructions of real-world crashes nor employed high biofidelity finite element human body models to analyze far-side occupant injuries. To address this gap, this study aims to conduct a series of kinematic reconstructions of real-world side-impact vehicle collision accidents. By extracting vehicle side-impact scenarios from accident data, numerical simulation analysis was employed to derive representative head and chest injury parameters for individual front-seat far-side occupants across all conditions. This approach, which employs detailed finite element (FE) human body models to mimic real-world biological responses, provides a pathway for a biomimetic understanding of injury. This will enable a comprehensive quantitative assessment of how diverse side-impact load conditions influence injury risks to the far-side occupant.

## 2. Methods and Materials

### 2.1. Accident Overview

**Figure 1:**
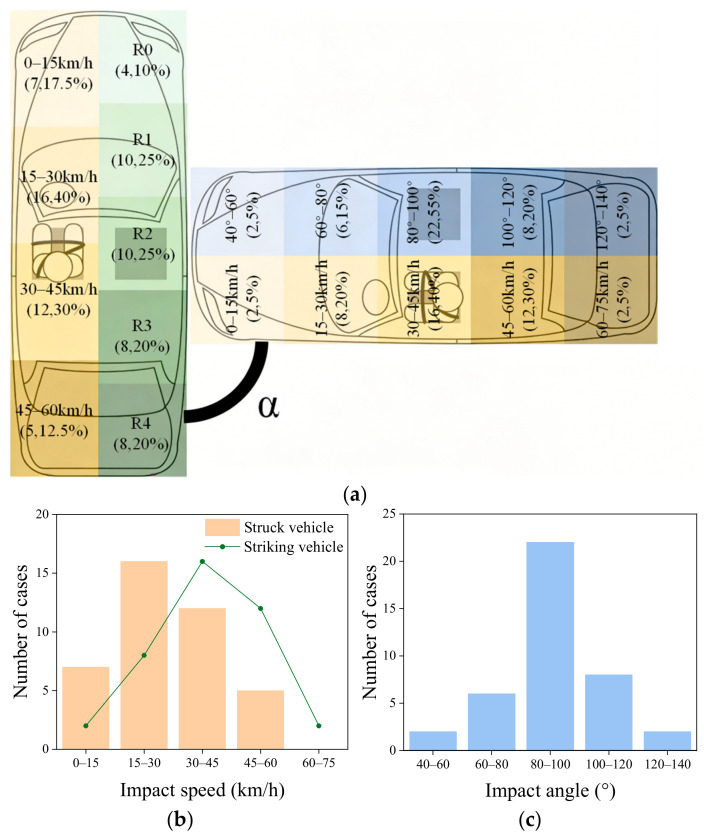
Accident information extracted from 40 accident cases: (**a**) general information; (**b**) impact speed; (**c**) impact angle.

The 40 vehicle side-impact accident cases used in this study were all sourced from publicly available data on online platforms (YouTube, Youku, and Bilibili). The criteria for selecting accident videos included the following: (1) video frame rate ≥ 25 fps; (2) one vehicle striking the side of another vehicle, capturing the entire motion sequence from pre-collision to post-collision; (3) vehicle brand and model identifiable. We preliminarily calculated the initial vehicle velocities from video footage and refined them through kinematic reconstruction [24]. Figure 1 shows the distribution of vehicle collision boundary conditions across 40 accidents. In Figure 1a, the yellow sections indicate the vehicle’s speed at the moment of collision, the green sections denote the collision region, and the blue sections represent the collision angle α of the vehicle.

### 2.2. Accident Reconstruction

**Figure 2:**
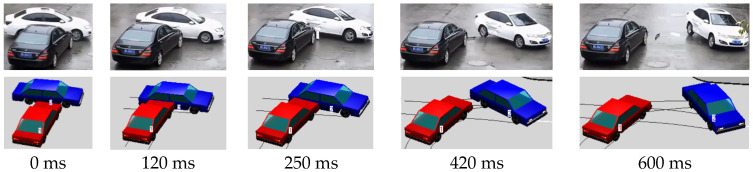
Comparison of actual vehicle kinematics and simulation results.

This study employed the accident reconstruction software PC-Crash 8.0 to reconstruct the accident scenario based on the preliminary vehicle boundary conditions obtained in the previous section [25] (including iterative calibration of friction coefficients and impact boundary conditions to match observed vehicle trajectories). Through iterative debugging, the vehicle’s motion trajectory was made to align closely with that in the video footage (Figure 2). The X and Y displacements and Z rotational velocity of the struck vehicle throughout the entire collision process were subsequently extracted. Additionally, we have listed the peak accelerations and angular velocities of the struck vehicles during the collisions in Table 1.

### 2.3. Finite Element Model

#### 2.3.1. Human Body Model

**Figure 3:**
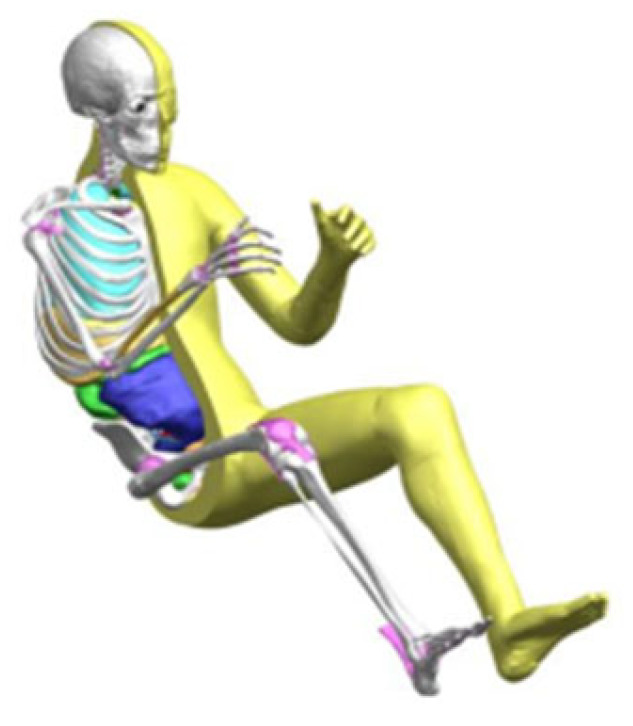
THUMS FE model.

This study used the THUMS 4.1 50th percentile adult male FE occupant model (Figure 3), jointly developed by Toyota Motor Corporation (Toyota city, Japan) and Toyota Central R&D Labs, Inc. (Nagakute City, Japan). This model has been widely used to simulate human kinematics and injury responses in traffic crashes, and its validation for far-side impact scenarios is among the most comprehensively documented in the literature [26,27,28]. Although newer versions (v5.0 and v6.0) exist, their validation under lateral rotational loading is not yet widely published, particularly for internal organ strain. Golman et al. [29] assessed the biofidelity of the THUMS model by comparing its biomechanical responses to cadaver test results.

#### 2.3.2. Vehicle Model

**Figure 4:**
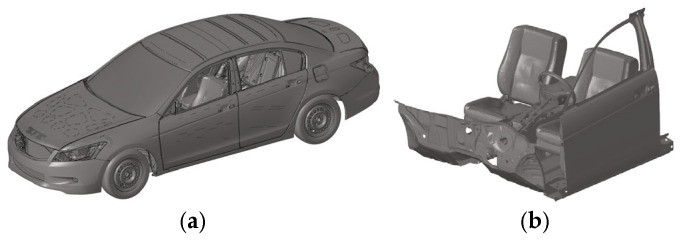
Vehicle FE Model: (**a**) Accord full-scale model, (**b**) simplified sled model.

We employed the full-scale vehicle FE model of the 2014 Honda Accord (Figure 4a), developed and shared by the National Highway Traffic Safety Administration (NHTSA), to conduct collision simulation analysis (https://www.nhtsa.gov/crash-simulation-vehicle-models, accessed on 14 November 2025). Given the high computational cost of FE simulations, this study simplified the aforementioned vehicle model to develop a sled crash model suitable for this research. This simplified model retains all vehicle components potentially involved in contact with the far-side occupant, including the chassis, steering wheel, seats, seat belts, center console, doors, etc. (Figure 4b). The average mesh size is approximately 10 mm.

#### 2.3.3. Human–Vehicle Coupling Model

**Figure 5:**
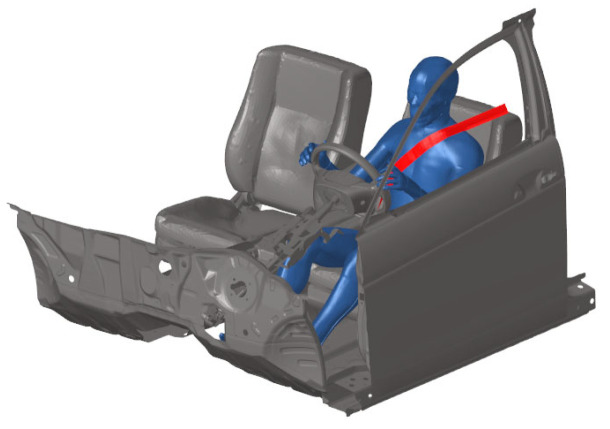
Human–vehicle coupled FE Model.

The THUMS occupant model was initially positioned using the marionette approach [30]. In this approach, seatbelt pretension and gravitational settling over 200 ms were applied to achieve a neutral seated posture consistent with SAE J4004 [31]. We verified the final joint angles against SAE J826 H-point data. We obtained the human–vehicle coupled model (Figure 5) through pre-simulation of the human body model’s posture and seatbelt. Then, the initial seating posture was adjusted through pre-simulation to comply with the SAEJ826 standard, and the seat belt pretension was set to 20 N [32]. Finally, the full kinematic response curve of the struck vehicle obtained during accident reconstruction was loaded onto the human–vehicle coupled model to simulate and analyze far-side occupant injuries. All FE simulations in this study were performed using LSDYNA R11.0 nonlinear explicit FE simulation software developed by LSTC (Livermore, CA, USA: http://www.lstc.com, accessed on 14 November 2025) [33], and each simulation ran for 600 ms.

### 2.4. Injury Assessment Criteria

#### 2.4.1. Head Injury Assessment Criteria

Head injuries can be classified into three main categories: (i) skull fractures; (ii) focal brain injuries, such as cerebral contusions; (iii) diffuse brain injuries. The Head Injury Criterion (HIC) is the most widely used indicator for assessing head injury, and it is closely associated with skull fractures and cerebral contusions [34,35]. HIC is calculated from the linear acceleration of the head center of gravity (CG) and its duration, using the following Formula (1). Moreover, this paper does not address optimization algorithms; all subsequent equations are solely employed for the assessment of injury risk.

(1) $$HIC={\left\{{\left(t_{2}-t_{1}\right)\left[\frac{1}{t_{2}-t_{1}}\int_{t_{1}}^{t_{2}}a(t)dt\right]}^{2.5}\right\}}_{max}$$

where *a*(*t*) represents the resultant acceleration of the head CG, measured in g; *t*_2_ − *t*_1_ denotes the duration of acceleration at which the HIC reaches its maximum value, set to either 15 ms or 36 ms.

Rotational motion of the head as a mechanism for brain injury was proposed back in the 1940s. The Brain Injury Criteria (BrIC) were proposed by Takhounts et al. based on dummy testing [36], and were primarily used at the macroscopic level to evaluate brain injury caused by rotational forces to the head. BrIC addresses the limitation of HIC, which does not account for head rotation, with the following calculation formula:

(2) $$BrIC=\sqrt{{\left(\frac{\omega_{x}}{\omega_{xC}}\right)}^{2}+{\left(\frac{\omega_{y}}{\omega_{yC}}\right)}^{2}+{\left(\frac{\omega_{z}}{\omega_{zC}}\right)}^{2}}$$

where *ω_x_*, *ω_y_*, *ω_z_* represent the angular velocities of the head CG, measured in rad/s; *ω_xC_*, *ω_yC_*, *ω_zC_* denote the standard angular velocities, with values of 66.3 rad/s, 53.8 rad/s, and 41.5 rad/s, respectively [34].

Maximum principal strain (MPS) serves as an indicator for assessing the severity of brain tissue injuries such as cerebral contusion and diffuse axonal injury (DAI) [36]. The cumulative strain damage measure (CSDM) is another important indicator for evaluating brain injury, represented by the proportion of brain tissue volume subjected to principal strain exceeding a certain threshold [37]. In this study, CSDM_0.15_ (i.e., with a principal strain threshold of 0.15) was employed to analyze traumatic brain injury.

#### 2.4.2. Chest Injury Assessment Criteria

The effective plastic strain of ribs serves as a critical indicator for assessing rib fracture risk. Through in vitro testing of human rib specimens, Kemper et al. determined that the effective plastic strain threshold for cortical rib fractures is 0.0271 [38]. The viscous criterion (VC) is primarily used to assess thoracic soft tissue injury during high-speed collisions [39], with its calculation dependent on instantaneous chest velocity and chest compression percentage [30]:

(3) $$VC=\frac{d\left[D\left(t\right)\right]}{dt}\times \frac{D\left(t\right)}{D\left(0\right)}$$

where *D* represents the chest cavity thickness prior to impact, while *D*(*t*) denotes the relationship between chest cavity thickness and time, measured in m; *VC* is measured in m/s.

This study also examined visceral injury in the thoracic cavity of the far-side occupant. Cavanaugh et al. [40] found that the strain threshold for heart injury was 0.3; according to Stitzel et al. [41,42], the strain threshold for lung injury was 0.35, and the strain thresholds for liver, kidney, and spleen injury were also 0.3 [43,44].

## 3. Results

### 3.1. Far-Side Occupant Kinematic Response

**Figure 6:**
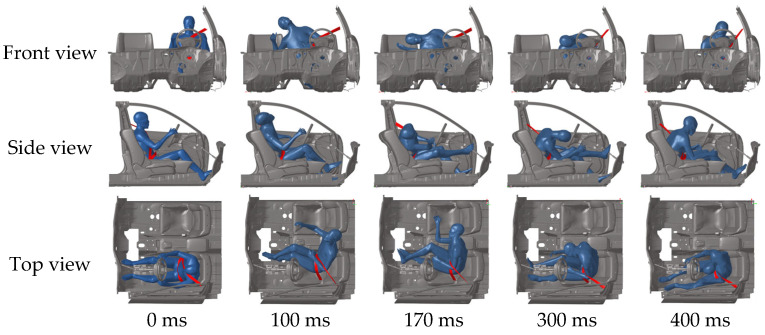
Kinematics response of the far-side occupant for one case.

**Figure 7:**
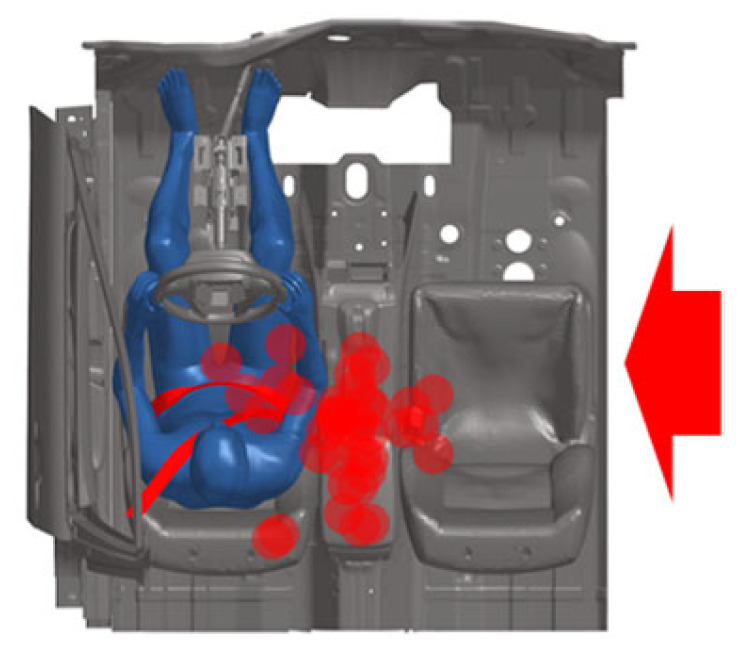
Maximum head displacement of the far-side occupant in all cases. Red arrow: the direction of impact. Circles: the maximum displacement positions of the far-side occupant’s head in all cases.

Figure 6 shows the kinematic response of the far-side occupant in a case study. The struck side of the vehicle undergoes rotational and lateral displacement upon collision, causing the far-side occupant to shift laterally toward the impact side. Maximum lateral head displacement occurs at 170 ms. Concurrently, the collision causes the struck vehicle to decelerate. Due to inertia, the far-side occupant leans forward (300 ms) before being restrained by the seatbelt (400 ms). Similar movement patterns are observed in other cases, consistent with Golman et al. [29]. The maximum displacement positions of the far-side occupant’s head in all cases are indicated by the red dot in Figure 7. During lateral movement, the occupant’s head also tends to move forward or backward. In some instances, substantial vehicle rotation may result in no lateral movement, with the head moving only forward.

### 3.2. Head Injury

**Figure 8:**
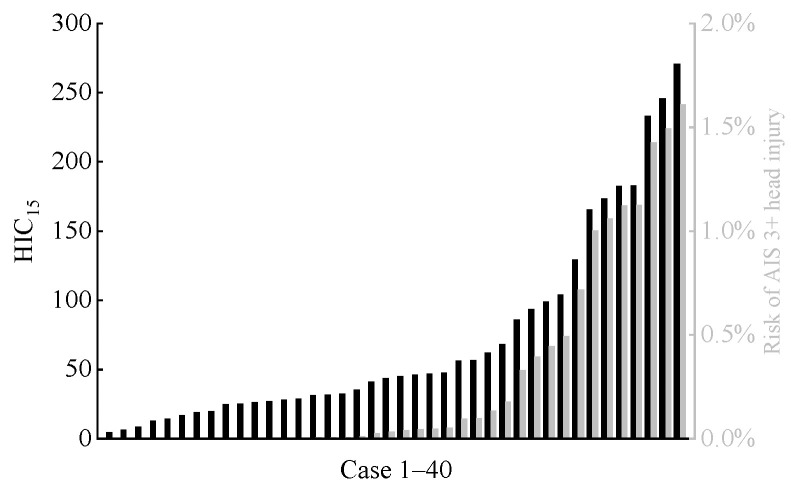
Predicted HIC_15_ (black columns) for far-side occupant and corresponding risk (gray columns) of AIS 3+ head injury across all cases.

**Figure 9:**
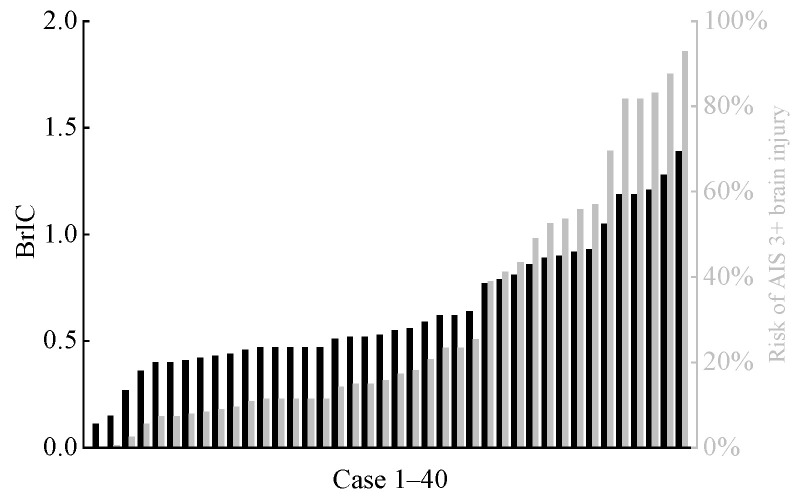
Predicted BrIC (black columns) for far-side occupant and corresponding risk (gray columns) of AIS 3+ brain injury across all cases.

The calculated HIC15 values for the far-side occupant in each accident scenario are shown in Figure 8. Additionally, based on the relationship between HIC15 and the risk of severe head injury (AIS 3+) as described in the literature [45,46], we calculated the risk of severe head injury for all far-side occupants. Overall, in the accidents analyzed in this study, the HIC15 values for the heads of far-side occupants were at relatively low levels, with the risk of AIS 3+ head injury remaining below 2% in all cases. BrIC is associated with the occurrence of traumatic brain injury [36,37]. Figure 9 shows the distribution of predicted BrIC values for far-side occupants across all cases, along with their corresponding AIS 3+ brain injury risks. These risks were derived from the BrIC-AIS 3+ brain injury risk relationship curve described in Takhounts et al. [36]. In 11 cases, the risk of far-side occupants sustaining AIS 3+ brain injury exceeded 50%.

**Figure 10:**
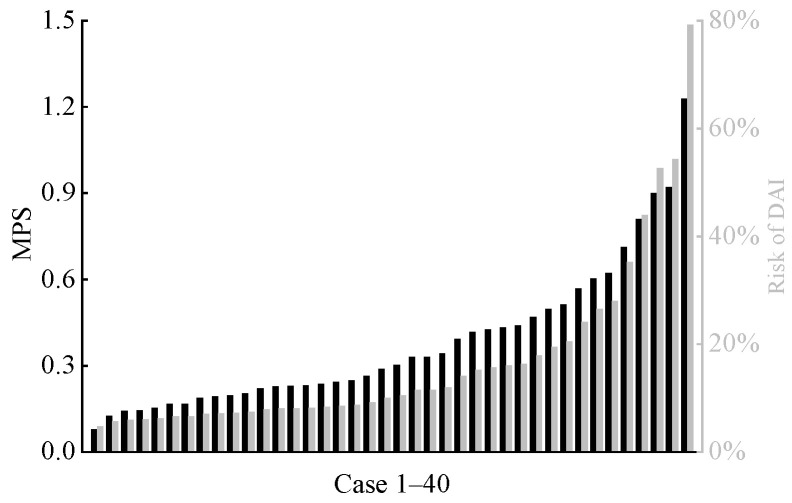
Predicted MPS (black columns) for far-side occupant and corresponding risk (gray columns) of DAI across all cases.

**Figure 11:**
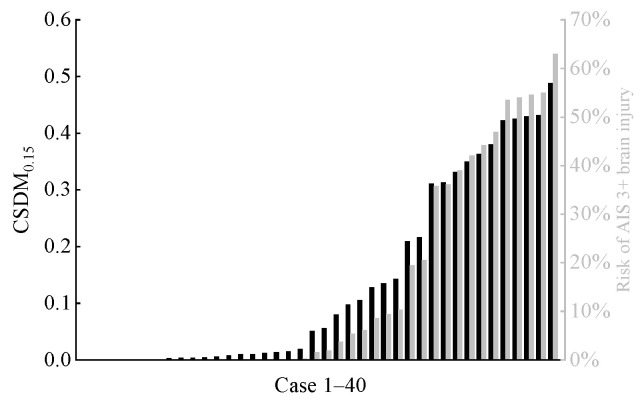
Predicted CSDM_0.15_ (black columns) for far-side occupant and corresponding risk (gray columns) of AIS 3+ brain injury across all cases.

Similarly, we calculated two brain tissue deformation-based injury criteria (MPS and CSDM0.15). The corresponding risks of DAI and AIS 3+ traumatic brain injury were determined by referencing established relationship curves for each criterion [36,37], as shown in Figure 10 and Figure 11. In all cases, only three cases showed a risk exceeding 50% for the far-side occupant to sustain DAI, and five cases indicated a risk exceeding 50% for the far-side occupant to sustain AIS 3+ traumatic brain injury.

### 3.3. Chest Injury

**Figure 12:**
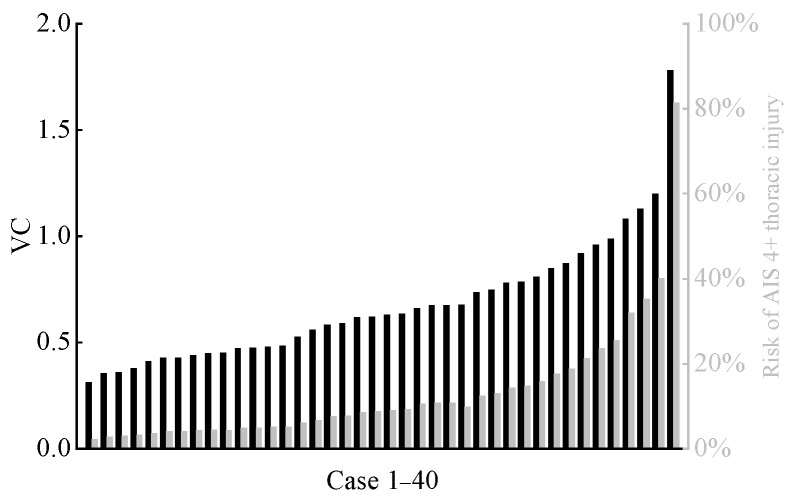
Predicted VC_max_ (black columns) values of far-side occupants and corresponding risk (gray columns) of AIS 4+ chest injury across all cases.

**Figure 13:**
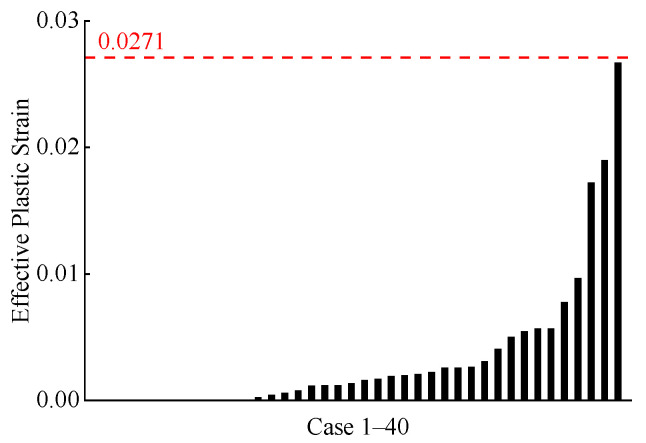
Predicted effective plastic strain of the rib cortical bone of the far-side occupant across all cases. The black dashed line represents the rib fracture threshold described in the literature [38].

The distribution of calculated VCmax for the far-side occupant across all cases is shown in Figure 12. We also calculated injury risk based on the VC-AIS 4+ severe chest injury risk relationship curves reported in the literature [30,47,48]. Although only one case predicted a risk exceeding 50%, the majority of predicted VCmax fell between 0.5 and 1.0. This indicates that the far-side occupant still faces a moderate to low risk of chest injury [47]. The effective plastic strain of the far-side occupant’s rib cortical bone is shown in Figure 13. In the vast majority of cases, the plastic strain values of the rib cortical bone were below the threshold of 0.0271 [38,49], indicating a low probability of rib fracture.

**Figure 14:**
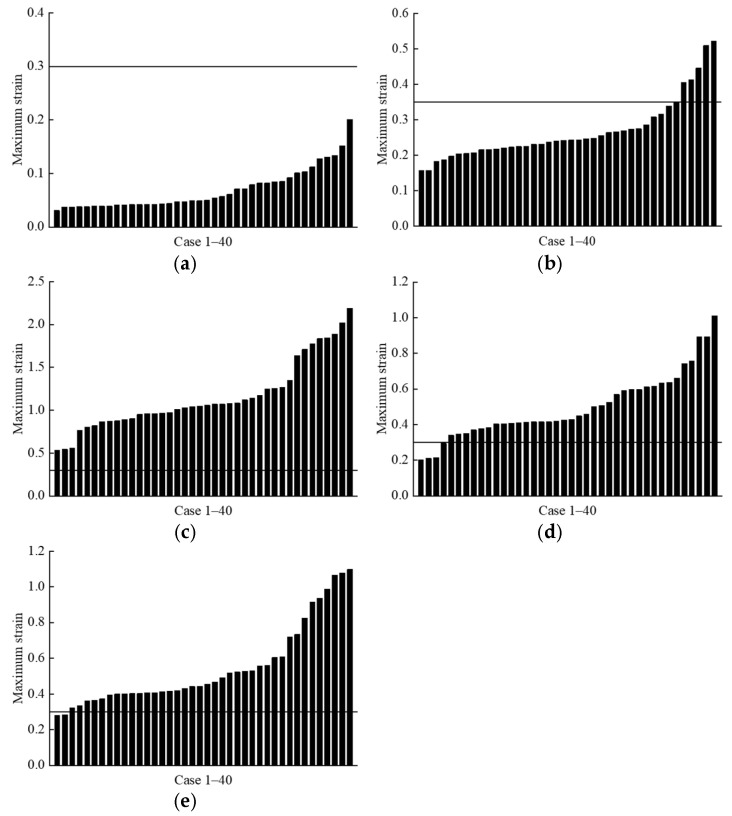
Predicted strain distribution of the internal organs of the far-side occupant across all cases. The black lines indicate the injury thresholds described in the literature [41,43,50]: (**a**) heart; (**b**) lung; (**c**) liver; (**d**) kidney; (**e**) spleen.

The distribution of maximum strain values for the visceral organs of the far-side occupant across all cases is shown in Figure 14. In all cases, the strain experienced by the occupant’s heart was significantly below the threshold of 0.3 reported in the literature [50] (Figure 14a). In 7 out of 40 cases, the strain experienced by the far-side occupant’s lungs approached or exceeded the threshold of 0.35 [41] (Figure 14b). For the liver, kidney, and spleen, strain values exceeded the literature thresholds in nearly all cases, particularly for the liver [43] (Figure 14c–e).

### 3.4. The Effect of Load Boundary Conditions on Far-Side Occupant Injury

Through the analysis of the aforementioned far-side occupant injury results, this study found that far-side occupant injury primarily concentrates in chest soft tissue injuries, specifically reflected in VC_max_ and the peak strains of the liver, kidney, and spleen. Therefore, we conducted further observations on the effect of vehicle load boundary conditions on the injury parameters for the far-side occupant.

**Figure 15:**
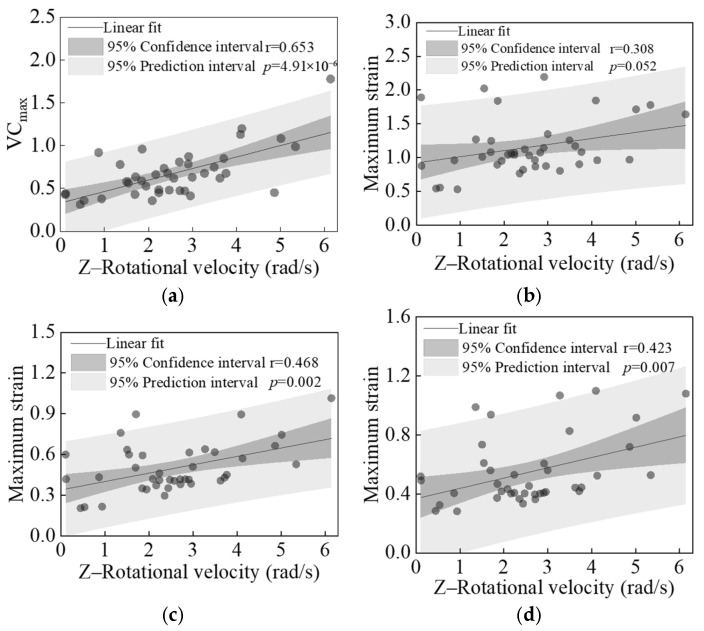
Linear regression results between the peak Z-direction rotational velocity of the struck vehicle and four analyzed injury indicators: VC_max_ (**a**), maximum strains of liver (**b**), kidney (**c**), and spleen (**d**).

Figure 15 shows the linear regression results between the peak Z-direction rotational velocity of the struck vehicle in each case and the calculated values of four primary injury indicators. All analyzed chest injury indicators (VC_max_ and peak strains of liver, kidney, and spleen) showed significant positive correlations with the *Z*-axis rotational velocity of the struck vehicle (*p* = 0.052 for liver, *p* < 0.05 for other parameters, and r > 0 for all parameters), particularly VC_max_ (*p* = 4.91 × 10^−6^).

**Figure 16:**
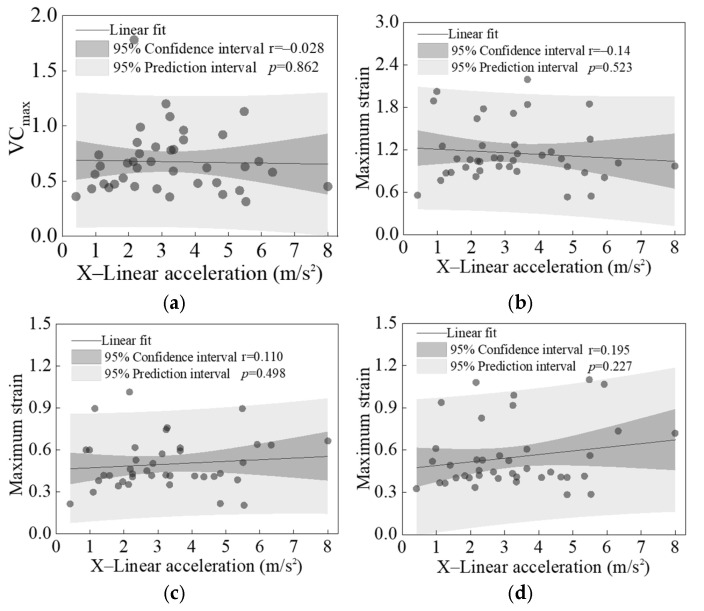
Linear regression results between the peak X-direction linear acceleration of the struck vehicle and four analyzed injury indicators: VC_max_ (**a**), maximum strains of liver (**b**), kidney (**c**), and spleen (**d**).

**Figure 17:**
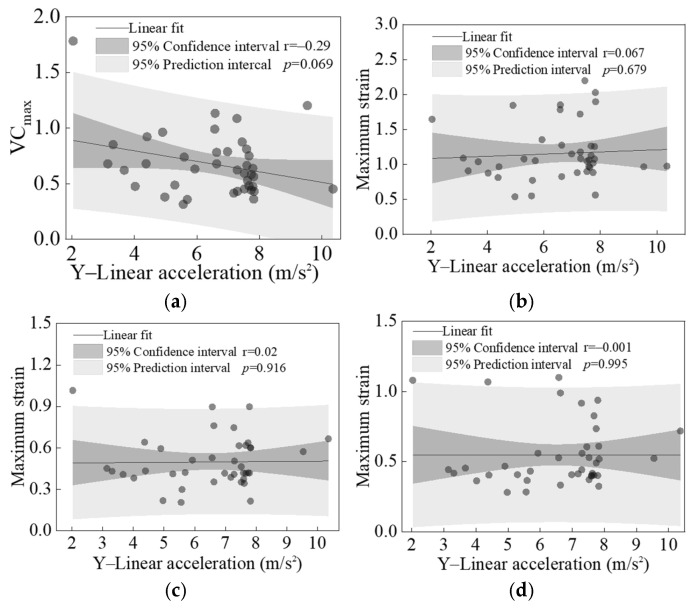
Linear regression results between the peak Y-direction linear acceleration of the struck vehicle and four analyzed injury indicators: VC_max_ (**a**), maximum strains of liver (**b**), kidney (**c**), and spleen (**d**).

Figure 16 and Figure 17 present the linear regression results between the peak linear accelerations in the X and Y directions of the struck vehicle and the calculated values of four primary injury indicators across all cases. Except for the *p*-value between the peak Y-direction linear acceleration and VC_max_, which was close to 0.05 (See Figure 17a), all other *p*-values were significantly greater than 0.05. This indicates that no significant correlation exists between the peak X- and Y-direction linear accelerations of the vehicle and any of the injury indicators.

## 4. Discussion

In this study, the predicted maximum HIC_15_ value for a far-side occupant’s head was 270.73, corresponding to an AIS 3+ severe head injury risk of only 1.6% (see Figure 8). Given that all far-side occupants’ heads in this study underwent inertial motion without hard contact with vehicle components and thus did not experience significant linear acceleration, the overall low HIC_15_ values—calculated solely from head CG linear acceleration and the duration—are reasonable. In contrast, BrIC calculations indicated a higher risk. Although the risk was below 50% in most cases, 13 cases showed risks exceeding 40%, including 5 cases above 60% and a maximum of 92.9% (see Figure 9). This finding is consistent with the study by Forman et al. [12]. In the present study, due to the rotational motion of the vehicle during the crash, some far-side occupants experienced a combined head motion involving rotation about the *Y*-axis and forward flexion, which substantially elevated their risk of severe traumatic brain injury. The brain injury risks predicted by MPS and CSDM_0.15_ were generally lower. However, similar to the BrIC analysis, there were still individual cases where the corresponding brain injury risks for MPS and CSDM_0.15_ exceeded 50%, and even approached 80%.

Compared to head injury, the risk of chest injury for far-side occupants is significantly higher. In all cases, the calculated effective plastic strain of the far-side occupant’s cortical ribs was well below injury thresholds, indicating a low rib fracture risk. However, analysis of the VC_max_ revealed a higher risk of chest soft-tissue injury, typically of moderate-to-low severity chest soft-tissue injury, for the far-side occupants. This aligns with the results for visceral organ injuries based on soft tissue strain analysis, particularly for the liver, kidney, and spleen. High organ strains must be interpreted alongside VC, which incorporates rate-dependent viscous effects that are critical to soft tissue injury. In fact, existing research [51] has confirmed that visceral injuries can occur through a viscous mechanism, which also serves as a critical factor in soft tissue injuries during side-impact collisions. Other studies [52,53] have further indicated that due to the inherently biomimetic, viscoelastic nature of the chest and abdomen, even minor deformations may lead to injury in internal organs. The elevated VC_max_ values observed in the chest region in this study suggest that visceral injuries are primarily induced by a viscous mechanism, aligning with the injury mechanisms described in the literature cited above.

This study examines the effects of three vehicle impact boundary parameters (X- and Y-direction linear acceleration, Z-direction rotational velocity) on far-side occupant head and chest injuries. Among these, the peak Z-direction rotational velocity showed significant positive correlations with VC_max_ and the peak strains of the liver, kidney, and spleen. In contrast, the peak X- and Y-direction linear accelerations showed no significant correlations with these injury indicators. Based on the injury characteristics of the head and thorax observed in far-side occupants in this study, as well as findings from existing research [5], it is evident that the rotational motion of the struck vehicle in side-impact scenarios is a key factor causing chest injury to the far-side occupant. Furthermore, brain injury associated with head rotational motion represents a critical focus for head injury protection for far-side occupants. These biomechanical insights, derived from a biofidelic simulation framework, must be considered in future developments of vehicle side-impact test protocols [54].

Unlike active safety systems focusing on human–machine cooperative control (e.g., driver-state-adaptive braking [55,56,57]), our work addresses passive safety during the crash phase, specifically the biomechanical response of occupants under complex multi-axial loading. Our key contribution lies in identifying vehicle rotational motion (a factor overlooked in current regulatory tests) as a critical driver of internal organ injury of the far-side occupants through viscous mechanisms, thereby informing the development of next-generation adaptive restraint systems [58,59] (e.g., rotation-sensing side curtains). Furthermore, from a biomimetic perspective, safety systems such as airbags could emulate the human body’s adaptive response to multi-axial loading. For instance, variable-stiffness side airbags could be deployed to enhance lateral head restraint when high rotational motion is detected.

This study focuses on inertial injury mechanisms (e.g., head swing, thoracic shear) in a single-occupant scenario and does not include multi-occupant interactions. Meanwhile, this study is also limited by the following: (1) use of only a 50th percentile male HBM, excluding females and elderly occupants; (2) fixed driver seating posture, without accounting for posture variability; and (3) reliance on a single vehicle geometry (Honda Accord), which may affect generalizability. Future work will expand to include multi-vehicle platforms, diverse anthropometric models, and advanced human body FE models.

Another key limitation of this study is the absence of modeled intrusion. Prior studies have reported higher HIC values in far-side collisions involving substantial compartment intrusion [60,61,62,63]. The relatively lower HIC values observed in our analysis may be because the real-world crash videos used predominantly represent low-to-moderate severity impacts with limited intrusion. Nevertheless, our findings regarding BrIC (indicative of shear-induced brain tissue injury) and VC (reflecting internal organ injury) remain valid in scenarios without significant intrusion. These results reveal an independent injury pathway that is overlooked by conventional intrusion-focused assessment approaches.

## 5. Conclusions

Based on the reconstruction and numerical analysis of real-world side-impact accidents, this study systematically investigated the injury risks of far-side occupants and their influencing factors, yielding the following key conclusions:

First, the head injury risk for far-side occupants is complex. HIC_15_-based analysis shows that the risk of AIS 3+ head injury from linear head acceleration is low, remaining below 2% in all cases. However, metrics such as BrIC, MPS, and CSDM_0.15_ reveal a high risk of severe traumatic brain injury in certain scenarios, with peak risk reaching 92.9%. This indicates that, for a single front-seat far-side occupant in a side impact, rotational vehicle motion, which causes angular head acceleration and brain tissue deformation, is the primary mechanism behind brain injury.

Second, chest injury exhibits a “low fracture risk, high soft-tissue injury risk” pattern. Analysis of rib effective plastic strain shows minimal rib fracture risk across all cases. In contrast, VC_max_ values indicate a high likelihood of chest soft-tissue injury. Strain assessments of internal thoracic organs (liver, kidney, and spleen) further confirm that injury thresholds are frequently exceeded, highlighting a critical vulnerability under rotational loading.

Finally, vehicle rotational velocity (peak Z-direction) showed significant positive correlations with VC_max_ and the peak strains of the liver, kidney, and spleen. In contrast, the peak X-direction and Y-direction linear accelerations of the vehicle exhibit no significant correlation with these injury indicators. This confirms that rotational motion of the vehicle is a key dynamic factor causing chest soft tissue and internal organ injuries to far-side occupants.

In summary, in a single-occupant scenario, far-side occupants experience distinctly different injury mechanisms compared to near-side occupants in side-impacts: head injury is primarily caused by rotational dynamics, while chest injury mainly involves soft tissues and internal organs due to vehicle rotation. These findings, based on biofidelic human body modeling, underscore the need to incorporate far-side occupant protection into future vehicle safety designs and assessment protocols (e.g., NCAP). Particular focus should be placed on mitigating injuries from rotational motion using safety systems that address multi-axial loading, thereby enhancing overall side impact safety.

## Figures and Tables

**Table 1:** Peak accelerations (in the X and Y directions) and angular velocities (in the Z direction) of the struck vehicles (Ang_V denotes angular velocity, X_Acc_ denotes the peak acceleration in the X direction, Y_Acc_ denotes the peak acceleration in the X direction).

Case ID	Ang_V (rad/s)	X_Acc_ (m/s^2^)	Y_Acc_ (m/s^2^)	Case ID	Ang_V (rad/s)	X_Acc_ (m/s^2^)	Y_Acc_ (m/s^2^)
1	1.68	−2.86	−7.30	21	5.34	−3.36	8.45
2	−2.36	−1.12	5.59	22	3.73	−2.26	−3.32
3	3.51	−2.33	7.68	23	0.04	−1.41	7.75
4	−2.59	−2.27	3.68	24	4.45	−3.52	7.29
5	−6.18	−2.17	−2.04	25	−3.75	−2.68	3.14
E	3.14	−5.35	7.18	26	1.86	−3.66	−4.90
7	1.71	−1.15	7.79	27	−2.94	−3.66	−7.46
8	−3.46	−5.92	−4.38	28	−0.24	−5.53	5.56
9	−2.45	−3.23	5.69	29	2.46	−4.65	−5.29
10	5.02	−8.11	7.42	30	2.49	−4.08	7.67
11	−4.14	−3.13	−9.54	31	1.53	0.31	7.83
12	−2.98	−1.25	−4.03	32	3.13	−3.35	6.99
13	2.27	−2.81	7.60	33	−0.56	−0.42	−7.83
14	−2.18	−1.97	−7.61	34	−4.1	−5.49	−6.59
15	−2.69	−1.58	−7.80	35	0.74	−4.84	4.98
16	−1.97	−2.135	−6.64	36	2.68	−4.84	−4.41
17	1.95	−1.83	−7.63	37	−3	−5.50	5.94
18	−0.13	−0.89	7.84	38	−1.36	−3.28	6.64
19	−2.24	−2.19	7.53	39	−1.5	−6.33	−7.75
20	−3.63	−4.36	7.30	40	−1.68	−3.38	−7.53

## Data Availability

The data presented in this study are available on request from the corresponding author due to the confidentiality agreements between the collaborative research units.
